# Supplementary material for: CaMK2rep: A Highly Sensitive Genetically Encoded Biosensor for Monitoring CaMKII Activity in Mammalian Cells
Source: Anal Chem. 2025 Sep 15;97(37):20275–90. doi: 10.1021/acs.analchem.5c03227 (PMC12461680; doi:10.1021/acs.analchem.5c03227)
Supplement: Supplementary file 1 [file ac5c03227_si_001.pdf]

SUPPORTING INFORMATION

Title:
CaMK2rep: A Highly Sensitive Genetically Encoded Biosensor for Monitoring CaMKII Activity in Mammalian Cells

Authors:
Elena Martínez-Blanco[1], Raquel de Andrés[2], Lucía Baratas-Álvarez[3] and F. Javier Díez-Guerra*

Author affiliations:
Group of Molecular Basis of Neuronal Plasticity
Departamento de Biología Molecular, Facultad de Ciencias
Centro de Biología Molecular (CSIC-UAM)
Universidad Autónoma de Madrid
Nicolás Cabrera, 1
28049 Madrid, Spain

[1]EMB ORCID: 0000-0003-3683-5096
[2]RdE ORCID: 0000-0003-3264-1008
[3]LBA ORCID: 0009-0003-3889-6834

***Corresponding author:**
F. Javier Díez-Guerra
E-mail: fjavier.diez@uam.es
Telephone: +34 91 196 4612
ORCID: 0000-0003-1707-4519
ResearcherID: K-9063-2014

Table of Contents:

SUPPORTING INFORMATION....S1
Figure S1. Design of CaMK2rep, a CaMKII activity reporter. S2
Figure S2. Schematic representation of CaMK2rep working principle and experimental workflow....S3
Figure S3. Quantitative analysis of CaMKII Activation in HeLa cells Using FRET-Based FRESCA biosensor. S4
Figure S4. nCaMK2rep3 specifically interacts with endogenous PSD-95 via the PSD95.FingR domain....S5
Figure S5. Neurogranin attenuates CaMKII activity both in basal and stimulated hippocampal neurons. S6
Table S1. Plasmids developed and used in this study. S7
Table S2. Plasmids obtained from Addgene. S8
Table S3.- Antibodies used in this study for WB and IFs. S9

Figure S1. Design of CaMK2rep, a CaMKII activity reporter.

A. SynP3myc is a synthetic DNA fragment that included 43 bp from mCherry C-terminal (red), 237 bp from a cDNA coding for amino acid sequence 543-620 from rat synapsin-1a (purple) with two copies of CaMKII-specific phospho-site 3 (underlined) and three consecutive copies of the myc tag (blue). Spot-NES sequence contains a Spot-tag (green), a nuclear export sequence (NES, orange) and 135 bp of the C-terminal sequence of mCherry (red). **B.** Immunofluorescence of HeLa cells transfected with CaMK2rep, with nuclei stained using DAPI. Images were acquired in the DAPI (ex378/52; em432/36) and GFP (ex474/27; em515/30) channels, with a 63X NA 1.4 objective in a Zeiss Axiovert 200M fluorescence microscope.

A

SynP3myc

CACCTCCCACAACGAGGACTACACCATTGTAGAGCAGTATGAAAGAGCCGAGGGTCGGCACAGCACAGGTGGGGCA
GCACGACCTCCGGCGTCCCCTAGCCCACAACGCCAAGCCGGTCCACCTCAGCCAATCCGACAGGCTAGTCAGGC
AGGGCCCGGTCCACCTAAAGTGTCAGGGGCGTCTCCCGGCGGGCAACAAAGACAGGGTCCTCCACAAAAACCGC
CGGGCCCAGCAGGTCCTATCCGACAGGCTTCCCAGGCTGGGCCTGGCCCAAGAACTGGCCCTCCCACGACGCAA
CAGCCCAGACCCGGATCCGGGGGTGAGCAAAAACTTATATCTGAGGAGGATCTTGGAGGTGGCGGAAGTGGAGGTG
GAGAACAAAAGCTAATATCAGAGGAGGATTTGGGAGGTGGCGGAAGTGGAGGTGGAGAGCAGAAGCTGATCTCTG
AAGAGGACCTCGGCGGAGGTGGATCCTAGTAAAAGCTTAAGTTTAAACCG

SPOT-NES

CCGCCACTGTGCTGGATATCTGCAGGAATGGTGAGCAAGGGCGAGGAGGACAACGGTACCATGGGTAGCCCCGATAGG
GTACGAGCTGTTAGCCACTGGAGCTCAGGCTCTAGTAGATTGCAGTTGCCTCCATTGGAAAGACTGACCCTCGATA
AGACTGAATTCGCTATCATAAAAGAATTTATGCGGTTTAAGGTTCATATGGAGGGGTCTGTTAATGGTCACGAGTT
CGAAATTGAGGGTGAAGGCGAGGGTCGCCCTTACGAGGGGACACAAACCGCCAAGCTGAAGGTGACCAAGGGC

B

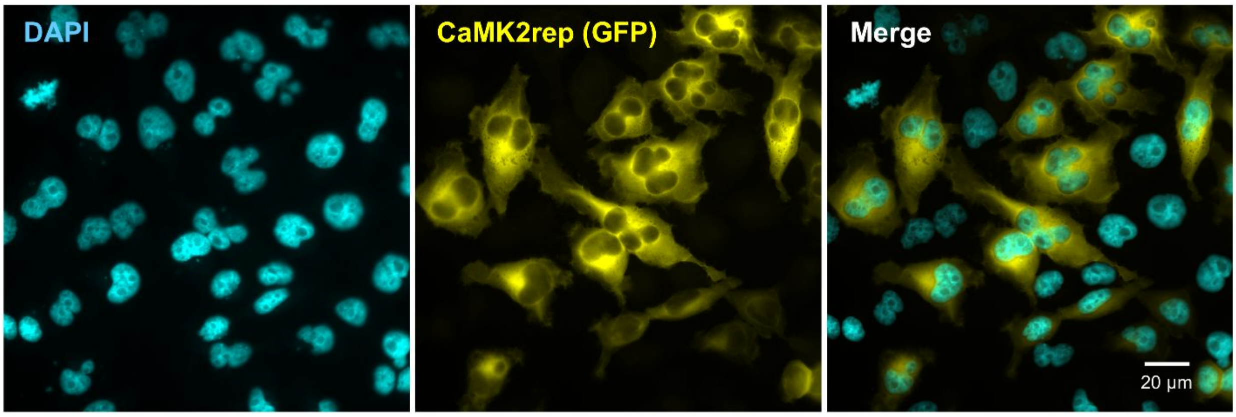

Figure S2. Schematic representation of CaMK2rep working principle and experimental workflow.

The 3D structures of CaMK2rep and nCaMK2rep were predicted using AlphaFold3 based on their respective amino acid sequences. The models suggest that both the synapsin phosphorylation sites (phosphosite-3) and the myc epitopes are accessible in what appears to be a randomly folded conformation. A) Schematic of CaMK2rep activation (phosphorylation) and detection by anti-phospho-Syn and anti-myc antibodies. B) Schematic of nCaMK2rep activation (phosphorylation) and detection by anti-phospho-Syn and anti-myc antibodies. Although each reporter has 3 replicates of the myc epitope, only one interaction anti-myc antibody is represented for clarity. C) Workflow diagram illustrating the experimental process, from treatment application in multiwell plates to the analysis of the phospho-synapsin/myc/CaMKII western blot bands intensity ratios. Icons are included to aid in visualizing the components shown in panels A and B.

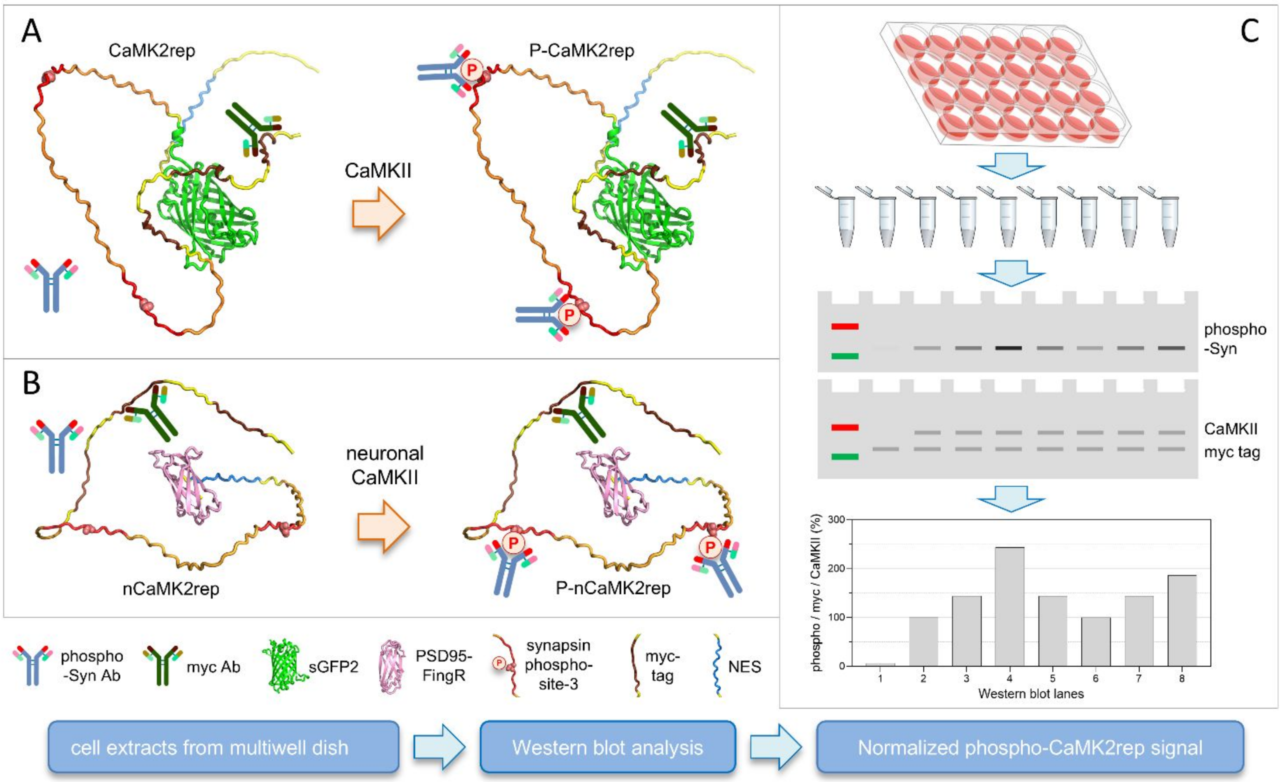

Figure S3. Quantitative analysis of CaMKII Activation in HeLa cells Using FRET-Based FRESCA biosensor.

HeLa cells seeded on coverslips were transfected with FRESCA and CaMKII 24 hours prior to analysis. On the day of the experiment, cells were preincubated for 15 minutes in Hanks' medium at 37ºC. Fluorescence imaging was then initiated in the CFP and YFP channels at 2-second intervals. After 1 minute of baseline acquisition, histamine (HA) was added at the concentration indicated in the histogram, and imaging continued for an additional minute. For each cell and time point, the ratio of mean fluorescence intensity between the CFP and YFP channels was calculated. These values were averaged across both baseline and HA-stimulated periods and normalized to each cell's baseline. A minimum of six recordings were performed for each condition.

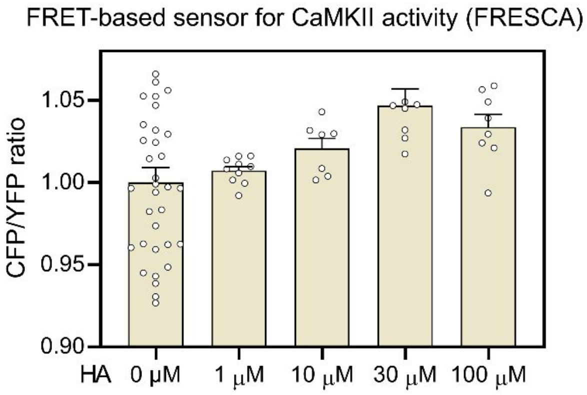

Figure S4. nCaMK2rep3 specifically interacts with endogenous PSD-95 via the PSD95.FingR domain.

Lysates of neurons (DIV16) expressing nCaMK2rep2 and nCaMK2rep3 were immunoprecipitated using anti-myc antibody. Non-infected neurons were utilized as controls (Cnt). A clear interaction of nCaMK2rep3 sensor with endogenous PSD-95 protein was observed, dependent on the PSD95.FingR intrabody domain. From this point, nCaMK2rep3 was routinely used to infect cultured neurons and we refer to it as nCaMK2rep.

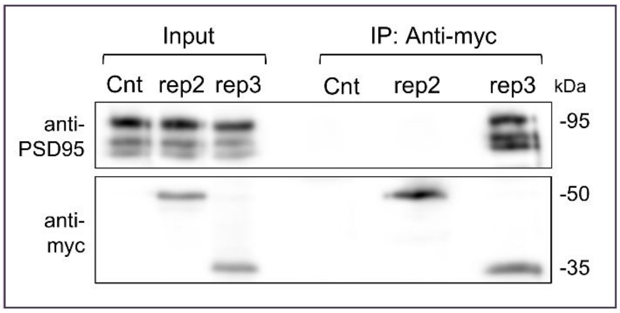

Figure S5. Neurogranin attenuates CaMKII activity both in basal and stimulated hippocampal neurons.

Hippocampal neurons expressing nCaMK2rep were infected at 7 DIV with AAVs to express Ng-wt, several Ng mutants, or to silence endogenous Ng. 15 DIV neurons were stimulated with 100 µM glutamate for 1 minute or left unstimulated. Basal and stimulated phosphorylation of nCaMK2rep was analyzed and normalized to the phosphorylation values obtained in the absence of Ng overexpression or silencing (mean ± SD, n=2).

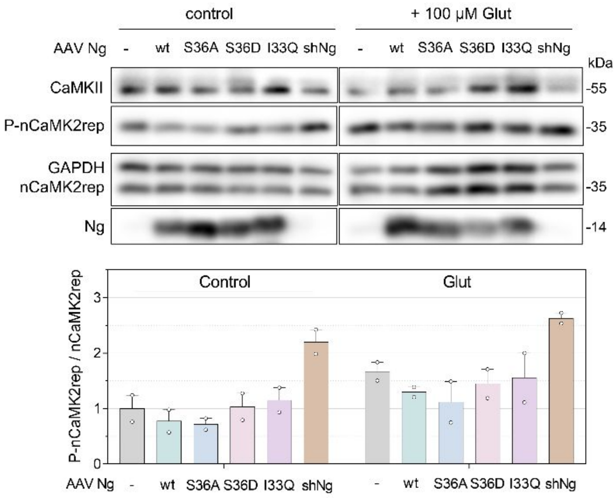

Table S1. Plasmids developed and used in this study.

Plasmids	Backbone	Insert	promoter	expression	tag
**CaMK2rep**	pcDNA3.1	SpotNES-sGFP2-SynP3-3xmyc	CMV	Mammalian	sGFP2, myc
**CaMKIIα**	pcDNA3<br>pLOX	CaMKIIα-3xmyc	CMV<br>synapsin	Mammalian<br>lentiviral	myc
**nCaMK2rep1**	pLOX	NES-SynP3-3xmyc	synapsin	lentiviral	myc
**nCaMK2rep2**	pLOX	NES-sGFP2-SynP3-3xmyc	synapsin	lentiviral	sGFP2
**nCaMK2rep3**	pLOX	PSD95.FingR-NES-SynP3-3xmyc	synapsin	lentiviral	myc
**nCaMK2rep4**	pLOX	PSD95.FingR-NES-sGFP2-SynP3-3xmyc	synapsin	lentiviral	sGFP2, myc
**tdTomato-P2A-jGCaMP8s**	pcDNA3	tdTomato-P2A-jGCaMP8s	CMV	Mammalian	tdTomato
**PKA-C-α-YFP***	pEYFP-N2		CMV	Mammalian	YFP
**PKCγ-YFP***	pEYFP-N2		CMV	Mammalian	YFP
**Ng**	pcDNA3<br>pAAV	Ng	CMV<br>CaMKIIα0.4	Mammalian<br>AAV2	
**Ng-S36A**	pcDNA3<br>pAAV	Ng-S36A	CMV<br>CaMKIIα0.4	Mammalian<br>AAV2	
**Ng-S36D**	pcDNA3<br>pAAV	Ng-S36D	CMV<br>CaMKIIα0.4	Mammalian<br>AAV2	
**Ng-I33Q**	pcDNA3<br>pAAV	Ng-I33Q	CMV<br>CaMKIIα0.4	Mammalian<br>AAV2	
**pAAV-shNg-mRuby2**	pAAV	shRNA targeting Ng	H1	AAV2	mRuby2
**pFΔ6****		AAV helper		AAV2	
**pRV1****		cap		AAV2	
**pH21****		rep-cap		AAV2	

* kindly donated by Dr Mark Dell'Aqua (Department of Pharmacology, University of Colorado, Denver, USA)
** kindly donated by Dr Hilmar Bading (Department of Neurobiology, Interdisciplinary Center for Neurosciences (IZN), Heidelberg, Germany

Table S2. Plasmids obtained from Addgene.

Plasmids	Addgene	Backbone	Insert	promoter	expression	tag
**CMV-tdTomato-P2A-paCaMKII**	#165431		tdTomato-P2A-paCaMKII	CMV	Mammalian	tdTomato
**CMV-tdTomato-P2A-paCaMKII K42M**	#165433		tdTomato-P2A-paCaMKII-K42M	CMV	Mammalian	tdTomato
**CaMKAR***	#205315	pcDNA3	CaMKAR	CMV	Mammalian	
**pAAV-CaMP0.4-FHS-paCaMKII-WPRE3**	#165429	pAAV	FHS-paCaMKII	CaMKIIα0.4	AAV2	FHS (Flag, HisX6, Strep)
**pAAV-CaMP0.4-FHS-paCaMKII(SD)-WPRE3**	#165430	pAAV	FHS-paCaMKII(SD)	CaMKIIα0.4	AAV2	FHS (Flag, HisX6, Strep)
**pAAV-shNg-mRuby2**	Modified from #92155	pAAV	shRNA targeting Ng	H1	AAV2	mRuby2
**pAAV-U6_CaMKIIa-mCherry**	#181875	pAAV	control shRNA	U6	AAV2	mCherry
**pCMVRδ8.74**	#22036		gag pol tat rev		lentiviral	
**pMD2.G**	#12259		VSV-G envelope		lentiviral	

* kindly donated by Dr. Jonathan M. Granger (Johns Hopkins University School of Medicine, Baltimore, USA)

Table S3.- Antibodies used in this study for WB and IFs.

Antibodies	Species	Antibody dilutions IF (cell culture)	Antibody dilutions WB	Reference
**CaMKIIα**	mouse		1:10.000	Millipore, 05-532 6G9
**cMyc**	mouse	1:5.000	1:30.000	Millipore, 05-724 4A6
**Phospho-synapsin-1 (S603)**	rabbit	1:2.000	1:20.000	Cell Signaling, D4B9I
**GAPDH**	mouse		1:30.000	Millipore, MAB374 6C5
**Ng**	rabbit	1:1.000	1:30.000	Millipore, AB5620
**PSD-95**	mouse		1:1000	Millipore, MAB1596 clone 6G6
**MAP2**	guinea pig	1:5.000		Synaptic Systems, 188004
